# Frequency Response Evaluation as Diagnostic and Optimization Tool for Pulsed Unipolar Plasma Electrolytic Oxidation Process and Resultant Coatings on Zirconium

**DOI:** 10.3390/ma16247681

**Published:** 2023-12-17

**Authors:** Evgeny Parfenov, Ruzil Farrakhov, Veta Aubakirova, Andrey Stotskiy, Rameshbabu Nagumothu, Aleksey Yerokhin

**Affiliations:** 1 Department of Materials Science and Physics of Metals, Ufa University of Science and Technology, 12 Karl Marx Street, Ufa 450008, Russia; 2 Department of Electronic Engineering, Ufa University of Science and Technology, 12 Karl Marx Street, Ufa 450008, Russia; frg1982@mail.ru (R.F.); veta_mr@mail.ru (V.A.); stockii_andrei@mail.ru (A.S.); 3 Department of Metallurgical and Materials Engineering, National Institute of Technology, Tiruchirappalli 620015, India; nrb@nitt.edu; 4 Department of Materials, University of Manchester, Oxford Road, Manchester M13 9PL, UK; aleksey.yerokhin@manchester.ac.uk

**Keywords:** plasma electrolytic oxidation, frequency response, in situ impedance spectroscopy, equivalent circuit, zirconium, zirconia, process control

## Abstract

This study aims to bridge various diagnostic tools for the development of smart plasma electrolytic oxidation (PEO) technologies. PEO treatments of commercially pure Zr were carried out using the pulsed unipolar polarisation (PUP) regime with frequency sweep in an alkaline phosphate-silicate electrolyte. Methods of in situ impedance spectroscopy and electrical transient analysis were used for the process diagnostics under the video imaging of the PEO. Two cutoff frequencies, 170–190 Hz and 620–650 Hz, were identified for the PEO-assisted charge transfer process. An equivalent circuit for the metal–oxide–electrolyte system under PUP PEO conditions was developed; from the capacitance values, two geometrical dielectric barriers were evaluated: a thinner 0.5–1 µm inner layer of the coating and a thicker 4–6 µm outer layer. These estimates were in agreement with the coating cross-sectional morphology. Based on comparing the results obtained using different techniques, the frequencies at which the uniform coatings with the best protective properties were formed were identified. For the selected electrolyte system and polarisation regime, these frequencies ranged from 2 to 5 kHz where the overall circuit reactance was minimal; therefore, the power factor was as close to one as possible. This opens the possibilities for the optimization of the pulsed PEO process and online control of unobservable surface characteristics, e.g., the thickness of the coating layers, thus contributing towards the development of smart PEO technologies.

## 1. Introduction

The surface engineering of zirconium is of interest for several important applications. In energy production, the cladding of fuel elements in light water nuclear reactors is made of Zr–Nb and Zr–Sn alloys that suffer from fretting corrosion and hydrogen embrittlement due to vibrations present in the reactor and contact with superheated (i.e., 400 °C) water and steam [1,2,3,4]. In the biomedical field, Zr–Nb and Ti–Zr–Nb alloys are being considered for orthopaedic and dental implants that require enhancements in bioactivity, biocompatibility and antimicrobial properties [5,6,7]. In optoelectronics, zirconium can be used as a substrate for the fabrication of photoluminescent materials based on rare-earth-doped zirconia films [8,9,10].

Chemical and physical vapour deposition, atomic layer deposition and other technologies are currently being considered for producing functional coatings on zirconium alloys [11,12,13,14,15]. Among them, plasma electrolytic oxidation (PEO) is a promising technique for the improvement of both the surface functional and protective properties of zirconium alloys [16,17,18,19,20,21]. However, an understanding of the phenomena of the Zr PEO process and the formalisation of the process mechanism have yet to be achieved because little attention is being paid to the PEO of zirconium alloys compared to that of aluminium, titanium and magnesium alloys [22,23,24]. The composition of the electrolyte and the electrical regime have a significant influence on the morphological and corrosion properties of PEO coatings [25,26,27]. The authors of [28] show the advantages of an electrolyte based on phosphates and silicates; these electrolytes can be modified with additives, for example, yttrium compounds [29,30] to stabilize the tetragonal phase and to achieve higher protective properties of the coating.

In situ impedance spectroscopy is a powerful tool for the investigation of the mechanisms underlying PEO processes; it is based on the estimation of the dynamic frequency response (FR) of the electrolyser impedance at a nonzero mean average current sustained by high-voltage polarisation. The methodology of FR measurements for diagnostics of PEO processes has been developed and discussed in detail elsewhere [31]. This methodology is being adopted by various research groups [32,33]. This approach bridges with electrochemical impedance spectroscopy, which allows equivalent circuits (ECs) to be applied for a formal description of electrolytic treatments [34,35,36]. Knowledge of equivalent circuits can help in understanding the transient effects that occur during the PEO treatments under pulsed polarisation and assist in optimizing the shape and frequency of the polarisation signal [30,37,38]. Furthermore, physically justified ECs can explain transitions between different stages in electrolytic plasma processes [38]. Previous studies have demonstrated the utility of this method for achieving a better understanding of electrolytic plasma processes operated under both DC [39] and pulsed bipolar polarisation [37]. However, in many cases, including the formation of PEO coatings on zirconium [40] and sometimes magnesium [41], pulsed unipolar polarisation (PUP) may be preferred, which necessitates the application of in situ impedance spectroscopy for studies of the PUP PEO processes.

Therefore, the joint application of the in situ impedance spectroscopy and transient analysis under video monitoring of the microdischarge appearance will provide insights into the PEO process mechanism on a Zr alloy. Consequently, this study aims to bridge these methods to develop an in situ diagnostic tool for smart and optimized PEO technologies operating at pulsed unipolar polarisation using the treatment of a Zr alloy in an alkaline phosphate-silicate electrolyte as an example.

## 2. Materials and Methods

**Figure 1:**
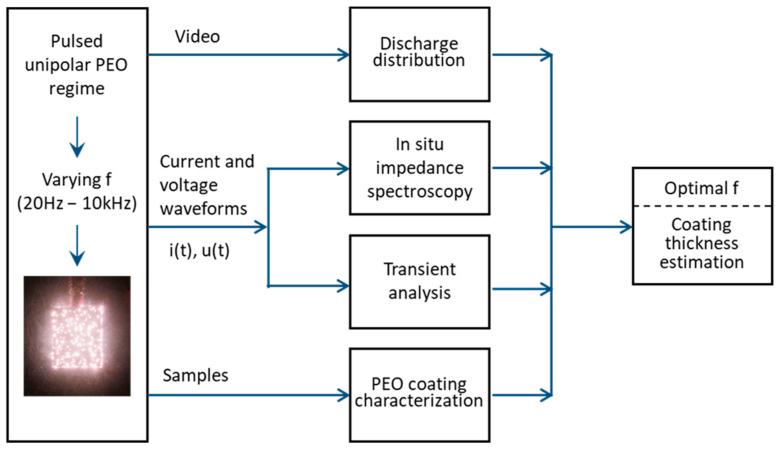
Schematic diagram of frequency response evaluation as a diagnostic and optimization tool for the PUP PEO of Zr.

Figure 1 presents a schematic diagram of the methods used in this research, which leverages frequency response evaluation as a diagnostic and optimization tool for the plasma electrolytic oxidation of zirconium under a pulsed unipolar regime. These methods are facilitated by a flexible, in situ adjustable PEO unit that can vary frequency and record the associated current and voltage waveforms. These waveforms are processed in two domains: the frequency domain using in situ impedance spectroscopy and the complementary time domain using transient analysis. Additionally, this study takes advantage of in situ video imaging to monitor the sample throughout the PEO process, followed by comprehensive image analysis. After the PEO process, the samples were analyzed using surface characterization techniques to evaluate the protective properties of the coating. The results of the in situ measurements and analysis were further compared with the coating properties to find strong correlations and to contribute to the PEO coating growth mechanism, process optimization and diagnostics.

### 2.1. Plasma Electrolytic Oxidation

**Figure 2:**
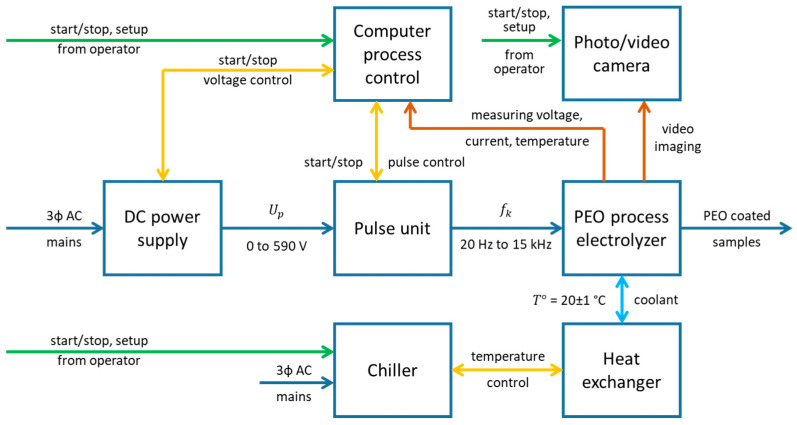
Schematic diagram of the experimental setup with frequency response capabilities for the PUP PEO.

Samples with a size of 15 mm by 11 mm were cut from a 0.8 mm thick sheet of commercially pure zirconium alloy (Zr-1wt%Nb). A schematic diagram of the experimental setup with frequency response capabilities for the PUP PEO is shown in Figure 2.

A 20 kW DC power supply provided the voltage, which can range from 0 to 590 V, to the pulse unit, which has a high power IGBT module to obtain voltage pulses at a programmed duty cycle and frequency. The resulting pulses were supplied to the anode and cathode terminals of the PEO process electrolyzer. The PEO process was performed in a 10-litre electrolyzer containing 4 L of aqueous electrolyte composed of (g L^−1^) 1 KOH, 2 Na_4_P_2_O_7_ and 2 Na_2_SiO_3_, all puris grade. The electrolyte temperature *T°* was maintained at 20 ± 1 °C by an external chiller. The sample was connected as the anode and placed in the center of the electrolyzer, and the heat exchanging coil was connected as the cathode. The computer process control and data acquisition system recorded the values of the voltage, current and electrolyte temperature. The video imaging was performed with an external camera.

A pulsed unipolar anodic polarisation signal with duty cycle *d* = 50% was applied. Initially, the voltage pulse magnitudes were ramped from 0 to 590 V for 45 s to provide a soft initiation of the PEO process. Further, the voltage pulse magnitude was kept constant at *U*_p_ = 590 V. Using the switching capabilities of the PEO equipment, the pulse frequency was swept from *f*_1_ = 20 Hz to *f*_K_ = 10 kHz in *K* = 15 steps, equally spaced in log scale (20, 28, 38, 62, 101, 164, 268, 435, 713, 1161, 1845, 3137, 4481, 7843 and 10,457 Hz) following the methodology described elsewhere [31]. With each step lasting for Δ*t* = 2 s, the total time taken by the whole sweep was *T* = 30 s, and the overall treatment time was *t*_PEO_ = *N*∙*T* = 7 min, with *N* = 14 sweeps attributed to each time window. Additional experiments were performed at constant frequencies of 62, 713 and 3137 Hz with the same treatment time of 7 min, following the previous studies.

### 2.2. Electric Signal Processing and Calibration

The instantaneous values of the voltage and current were recorded simultaneously, with a sampling frequency of *f_s_* = 0.5 MHz; this satisfies the Nyquist criterion. The voltage and current waveforms were acquired during the time frame *t_f_* = 100 ms every 1 s during the experiments, providing two recorded frames for each frequency step.

The impedance modulus $\left|Z_{k}\right|$ was obtained as a ratio of the RMS values of the corresponding voltage and current waveforms and recorded for a given frequency $f_{k},\;k\;\epsilon \;1\ldots K$ during a selected time frame:

(1) $$\left|Z_{k}\right|=\frac{U_{k}}{I_{k}},$$

where $U_{k}$ is the RMS value of the voltage waveform and $I_{k}$ is the RMS value of the current waveform.

The phase angle $\theta_{k}$ was estimated as a phase shift between the first harmonics of the pulses in the voltage and current waveforms during a selected time frame.

As for each frequency $f_{k}$, two time frames of the voltage and current were acquired, and mean values of the impedance modulus and phase angle were calculated and attributed with the selected frequency $f_{k}$ and time window $t_{n},\;n\;\epsilon \;1\ldots N$, resulting in the estimates $\left|Z(t_{n},f_{k})\right|$ and $\theta (t_{n},f_{k})$.

The in situ impedance spectra were obtained as discrete-time and discrete-frequency estimates of the complex-valued impedance:

(2) $$\underline{Z}(t_{n},j\cdot f_{k})=\left|Z(t_{n},f_{k})\right|\cdot e^{j\cdot \theta (t_{n},f_{k})}\;(\mathrm{where}\;j=\sqrt{-1}).$$

For compatibility with the impedance spectroscopy software, real and imaginary parts of these estimates were calculated:

(3) $$\underline{Z}(t_{n},j\cdot f_{k})=Z^{\prime }(t_{n},f_{k})+j\cdot Z^{\prime \prime }(t_{n},f_{k}).$$

The temporal resolution of the estimates equaled *T* = 30 s, and the frequency resolution was 0.2 decade; this gave 5 frequency points within a log frequency decade.

The spectra were validated by evaluating noise and non-linearity following the method described elsewhere [42] using FRAnalysis software (v. 3.0) [37] and then fitted to an appropriate equivalent circuit using ZView (Scribner Associates) software (v. 3.5).

The electrolyte resistance was estimated at *R_s_* = 130 Ω cm^2^ from the solution of a boundary value problem for the electric field distribution in a conductive electrolyte medium and kept constant in all the fits.

The voltage pulse transients following the current pulse shutoff were fitted using the MATLAB Curve fitting tool (v. 7.9) with exponential functions:

(4) $$u=U_{1}\cdot e^{-\left(\frac{t-t_{0}}{\tau_{1}}\right)}+U_{2}\cdot e^{-\left(\frac{t-t_{0}}{\tau_{2}}\right)}+C.\;$$

The coefficient of determination *R*^2^ for the fits was 0.97 and higher.

The frequency response measurement system was calibrated using an *R_s_*(*R_p_C_p_*) circuit with known nominal values of elements following the method described elsewhere [42]. The average errors for impedance modulus and phase angle were evaluated to be below 6% and 4 degrees, respectively, with the largest errors appearing at the highest frequencies.

### 2.3. Image Processing

The video recording of the PEO process was carried out using an Olympus E-PL camera at 30 frames per second. The exposure time was fixed at 1/1000 s and the aperture was set at f/3.5. A link to a typical video fragment of a frequency-swept PEO can be found in the Data Availability section of this paper. The video image processing was carried out using MATLAB software (v. 7.9) following the algorithm described below. The video sequence was divided into separate colour frames in JPEG format. Next, the frames were cropped to accommodate the images of microdischarges and some of the electrolyte bulk surrounding the sample. The cropped colour images were converted to 8-bit grayscale (with the brightness gradation from 0 to 255). Each frame was represented as a matrix containing the brightness values for the corresponding pixels. Then the brightness matrices were added up for the selected time duration (1 to 6 min). The resulting integral brightness values were normalised to the range [0, 1] to obtain the maximum glow intensity observed during a given experiment. For a visual representation of the glow intensity distribution, the normalised integral brightness values were displayed as a blue-yellow colour map.

### 2.4. Surface Characterization

The surface morphology was assessed using a Zeiss Gemini-300 scanning electron microscope (SEM) (Jena, Germany). The cross-sectional morphology was observed on mechanically polished samples following etching in a solution containing (mL) 10 HF, 15 HNO_3_ and H_2_O, with a thin layer of Pt deposited on the sample. The coating porosity was calculated from the SEM images using ImageJ software (v. 1.53). The coating thickness *h* was estimated from the cross-sectional SEM images; both the inner and outer layers of the coating were assessed.

The coating thickness *h* was also measured at the centre of the sample using a Defelsko Positector 6000 eddy current gauge (Defelsko, Ogdensburg, NY, USA) with an accuracy of ±0.1 µm. The surface roughness *R*a was measured at the edge (*Ra*_edge_) and the centre (*Ra*_centre_) of the sample using a TR 220 profilometer (TIME Group, Beijing, China). The integral parameter *Ra*_average_ was calculated as:

(5) $${Ra}_{average\;}=\frac{1}{S_{1}+S_{2}}(S_{1}\cdot {Ra}_{edge}+S_{2}\cdot {Ra}_{centre})$$

where *S*_1_ and *S*_2_ are surface areas of the edge and centre regions of the sample, respectively.

The corrosion behaviour of the coated samples was studied in 0.1 M LiOH solution using an Elins P-5X electrochemical station (Elins, Moscow, Russia). An Ag/AgCl in saturated KCl (*E*_0_ = 200 mV vs. SHE) and a Pt rod were used as reference and counter electrodes, respectively. The open circuit potential (OCP) was measured for 2 h to achieve a steady state value; then the potentiodynamic polarisation (PDP) tests were performed from –0.6 to +0.6 V around the OCP at the scan rate of 0.25 mV/s.

Usually, the PEO coatings exhibit anodic parts of the PDP curves that tend to show passivation behaviour, i.e., the current decreases with the potential increase [43]. Therefore, it is impossible to identify a Tafel slope in the anodic PDP curve, and the Stern–Geary equation cannot be used for the *i_corr_* calculation. Nevertheless, the cathodic parts indeed show Tafel slopes in the range within 300 mV from the free corrosion potential; consequently, the corrosion current density *i_corr_* was obtained from the cathodic Tafel parts of the PDP curves by finding the intersection of a tangent line to the cathodic part of the PDP curve with the level of the free corrosion potential *E*_corr_.

The samples were produced and characterized in at least 3 repetitions to obtain reliable statistical estimates of the coating properties.

## 3. Results and Discussion

### 3.1. Characteristics of the PEO Process under Swept and Constant-Frequency Polarisation

**Figure 3:**
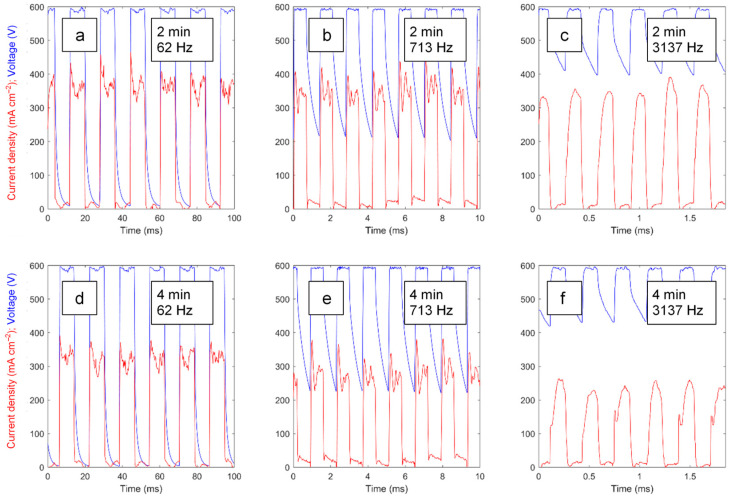
Typical voltage and current waveforms acquired during 2nd (**a**–**c**) and 4th (**d**–**f**) minute of frequency swept PEO treatment of Zr at characteristic frequencies 62 (**a**,**d**), 713 (**b**,**e**) and 3137 (**c**,**f**) Hz.

**Figure 4:**
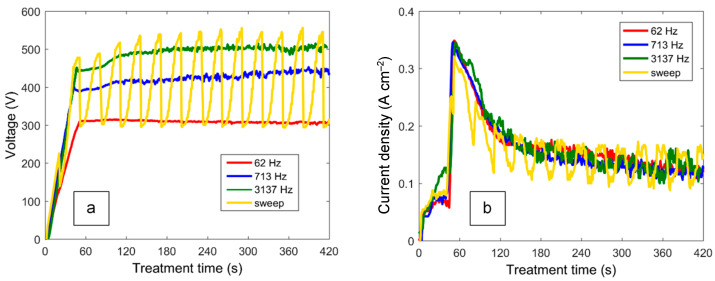
Evolution of DC values of voltage (**a**) and current density (**b**) during PEO treatments of Zr carried out in frequency-swept mode and at constant frequencies of 62, 713 and 3137 Hz.

Figure 3 shows the typical voltage and current waveforms obtained at characteristic frequencies during PEO treatments with frequency-swept polarisation. For the corresponding constant-frequency experiments, the waveforms appear to be similar. The current pulses exhibit peaks at the front edges, whereas the voltage pulses feature tails at back edges that are particularly noticeable at higher frequencies where the voltage does not fall to zero at the end of the pause between the pulses. These transients reflect the capacitive nature of the PEO as an electric load, and they become more pronounced with the treatment time, which is manifested in the DC values of the voltage (Figure 4).

For the pulses at 62 Hz, the DC voltage ramps up and stays at a constant level slightly higher than the product U_p_·d = 285 V. With the frequency increase, the DC voltage goes up since the waveforms become more affected by the gradual voltage fall during the pulse-off period. Due to this effect, the DC voltage values of the frequency-swept experiment oscillate with the cycle T = 30 s between the values of the constant-frequency experiments. The current densities exhibit lower values during the initial voltage ramp step (0–45 s); they were at a maximum at the beginning of the potentiostatic step (45–60 s) due to the ignition of the numerous microdischarges, but there was a gradual decrease (60–420 s) reflecting the growth of the PEO coating during the remainder of the treatment.

**Figure 5:**
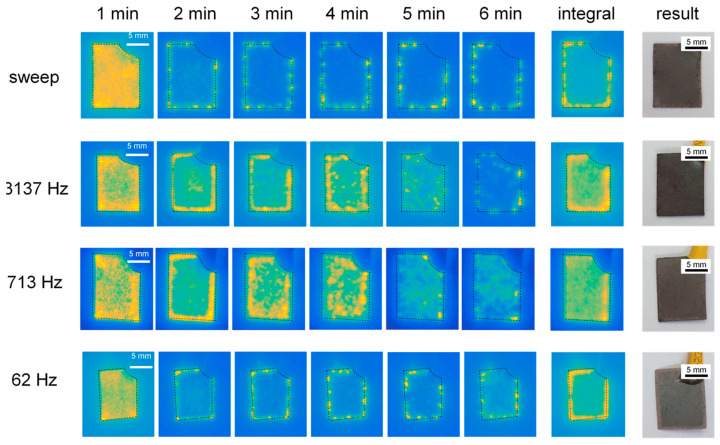
Microdischarge distribution during PEO treatments of Zr in the frequency sweep and constant-frequency modes at 62, 713 and 3137 Hz and resulting sample appearance after the treatments.

The microdischarge distribution during the studied PEO processes and the appearance of the resulting coated samples are shown in Figure 5. Dashed lines show the sample contour.

The appearance of the PEO microdischarges during the frequency sweep (at a treatment time of 4 min) can be seen in a video fragment which can be viewed via the link provided in the Data Availability section. As evident from the video, the microdischarges have different distributions over the sample surface at different frequencies. Violent microdischarges appear at the edges of the sample at the lower frequency (62 Hz). At the higher frequencies (713 and 3137 Hz), the microdischarges start migrating towards the sample centre, and the microdischarge distribution appears to be relatively uniform. As a result, the integral microdischarge distribution appears highly uneven at the lower frequency, whereas it looks relatively uniform at 713 and 3137 Hz. At the highest frequencies above 4 kHz, the microdischarges tend to become extinct. The resulting coating formed at 62 Hz features a burned morphology at the edges while the appearance of the coatings on the other samples is rather uniform; the coating obtained at 713 Hz still shows a burned edge effect.

The PEO process is usually self-levelling, i.e., a certain area of a sample that receives more current gets oxidized and, therefore, cannot receive more current, thus redistributing it to neighbouring areas. Nevertheless, this is not the case for the PUP PEO of Zr in the alkaline silicate-phosphate electrolyte, and the secondary current density distribution in the surface layer in this case strongly depends on the primary current density distribution in the electrolyte. This effect has a strong frequency dependency.

The coating formed during the frequency-swept experiment shares the above features, albeit with a less burned morphology at the edges, reflecting a more uniform integral microdischarge distribution than the coating formed at the lowest constant frequency.

### 3.2. In Situ Impedance Spectra of the PUP PEO Process

**Figure 6:**
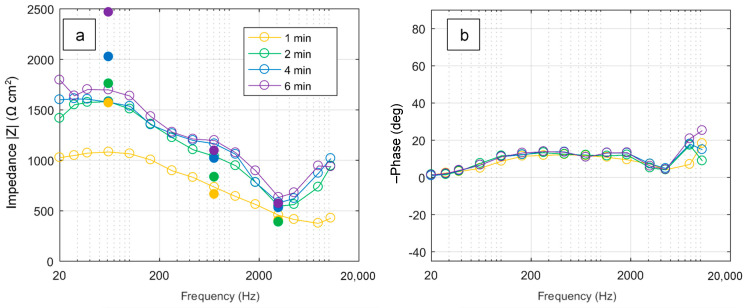
Evolution of impedance modulus (**a**) and phase angle (**b**) during PEO treatments of Zr. Solid lines and dots represent frequency sweep estimates and constant-frequency data, respectively.

Figure 6 shows the evolution of the impedance spectra with the PEO treatment time, with blank data points and lines denoting the impedance estimates derived from the frequency-swept PEO process and filled data points denoting those carried out at constant frequencies. The impedance modulus is high at frequencies below 160 Hz but then decreases to a minimum at about 3 kHz, beyond which it starts to increase again. The modulus increases with time following the coating growth, and a plateau starts developing at about 1 kHz after 2 min of the treatment. 

Constant-frequency experiments show similar impedance modulus behaviour, although the values at the lowest frequency are higher than those derived from the frequency-swept run. The phase angle is located in the capacitive response quadrant and reaches a maximum at 200 to 300 Hz but then decreases to almost zero at 3 to 5 kHz before increasing again.

**Figure 7:**
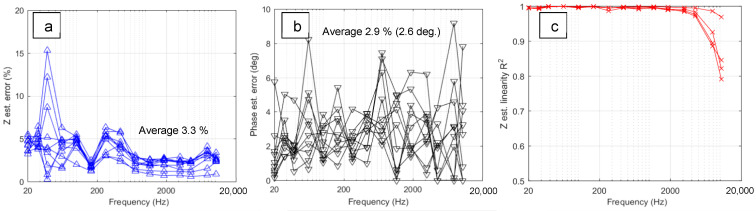
Noise and linearity assessment of in situ impedance spectra for the PEO treatment of Zr: impedance modulus error (**a**), phase angle error (**b**) and degree of linearity R^2^ (**c**).

The estimation of the noise and non-linearity using FRAnalysis software (v. 3.0) [37] is shown in Figure 7. The mean average errors of the impedance modulus and phase angle appear within 5%, and the average system linearity is estimated to be above 90%. The errors increase at lower and higher frequencies due to limitations in the number of signal cycles and sampling frequency, respectively; the system linearity decreases with frequency due to the latter effect. This assessment indicates that in situ frequency response estimates can be considered reliable for the investigation of the PEO processing of Zr in the PUP regime (with the available equipment) in the frequency range from 20 Hz to 4 kHz where the system linearity is over 95%.

**Figure 8:**
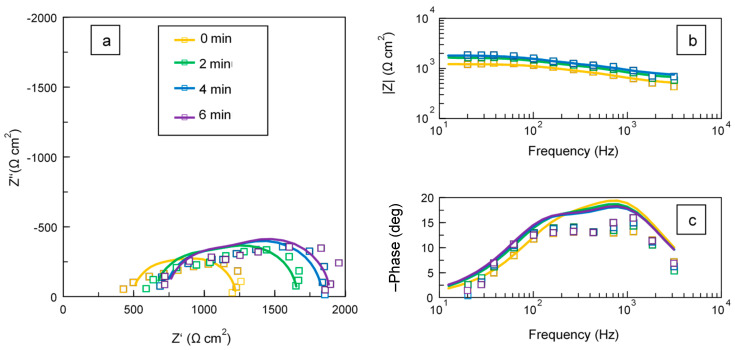
Evolution of complex (**a**) and Bode (**b**,**c**) fitted plots of in situ impedance spectra for the PEO treatment of Zr.

**Figure 9:**
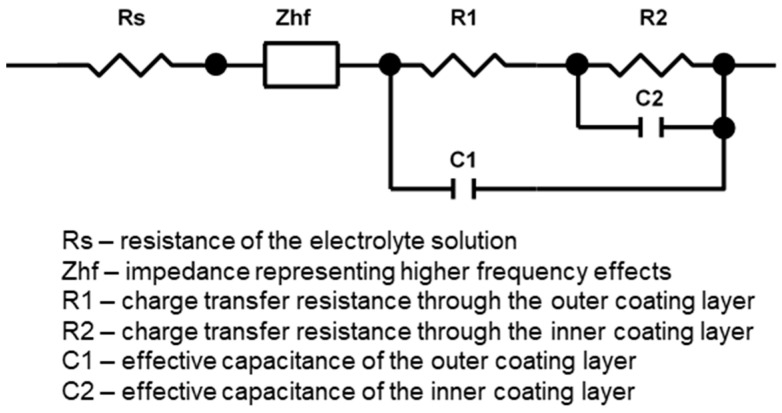
Equivalent circuit used to fit the in situ impedance spectra of the PEO process on Zr.

Figure 8 shows complex and Bode plots of in situ impedance spectra of the studied PEO process fitted to the equivalent circuit (EC) presented in Figure 9, with values of the circuit elements summarised in Table 1. The adopted EC presents a two-step ladder structure reflecting two time constants: one corresponding to the frequencies between 100 and 200 Hz and the other to the band between 500 and 5000 Hz. At these frequencies, the phase angle plot significantly changes its slope, indicating the presence of a cutoff frequency. The impedance complex plots feature two *R*–*C* loops representing relaxation processes with time constants τ_1_ = *R*_1_·*C*_1_ and τ_2_ = *R*_2_·*C*_2_ and cutoff frequencies *f_c_*_1_ = 1/(2πτ_1_) and *f_c_*_2_ = 1/(2πτ_2_) that are analysed further.

Since the data for the frequencies above 4 kHz were considered to be unreliable, three experimental points at 4481, 7843 and 10,457 Hz were excluded from the fits. Nevertheless, the input of the higher-frequency impedance must be taken into account. Therefore, it was included in the EC as a resistance of the *Z_hf_* element connected in series with *R_s_*; both were evaluated as a single resistance in the ZView software (v. 3.5) during the fit.

### 3.3. PEO Coating Characteristics and Properties

**Figure 10:**
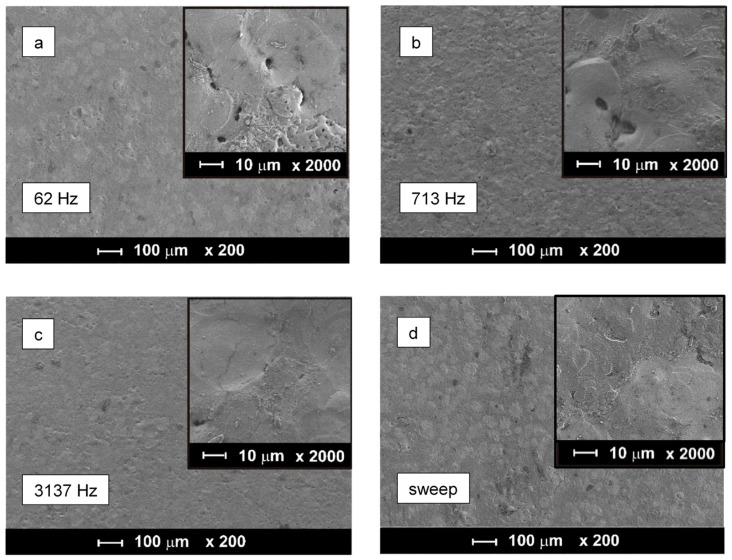
Surface morphologies of PEO coatings on Zr produced in the frequency sweep mode (**d**) and at the constant frequencies of 62 Hz (**a**), 713 Hz (**b**) and 3137 Hz (**c**) (sample centre).

**Figure 11:**
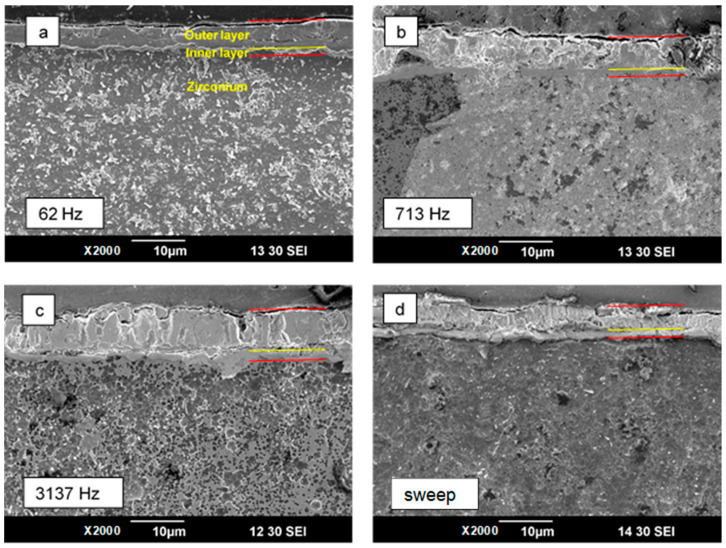
Cross-sections of PEO coatings on Zr produced in the frequency sweep mode (**d**) and at the single frequencies of 62 Hz (**a**), 713 Hz (**b**) and 3137 Hz (**c**). Red lines show the coating thickness, yellow line indicates the inner layer.

Figure 10 shows surface SEM images of the PEO coatings on Zr taken in the central areas of the samples treated under frequency-swept and constant-frequency polarisation. The coatings present a typical porous structure produced by numerous melting–solidification cycles induced by microdischarges accompanying the oxidation of zirconium. The most uniform and smooth coating was produced by the PEO treatment at 3137 Hz. Lower frequencies led to coarser porosity and uneven surface morphology around microdischarge craters. For the coating produced at 62 Hz, these features resemble a “leopard skin” pattern under lower magnification. A similar morphology was also observed in the coating produced by the frequency-swept experiment, although the pore openings appeared to be more compact. The cross-sections of the PEO coating on Zr are presented in Figure 11; all the coatings exhibit a porous outer layer 5–6 µm thick and a compact inner layer 1–2 µm thick.

**Figure 12:**
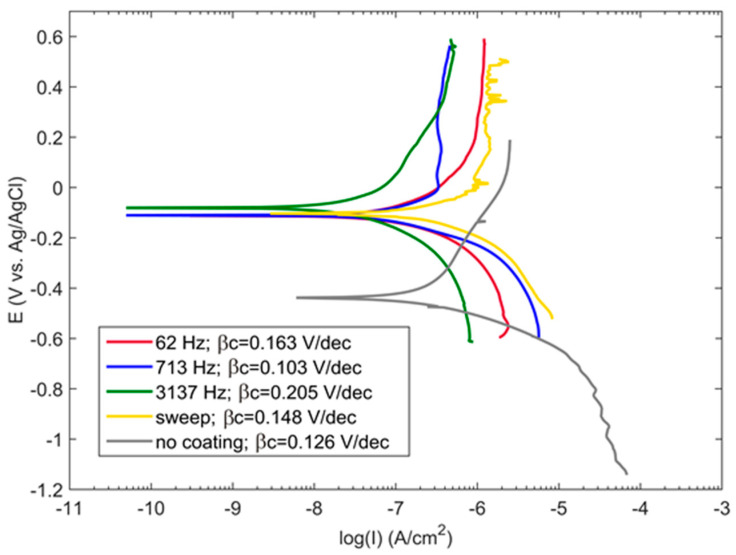
PDP curves obtained in 0.1 M LiOH solution for the PEO coatings on Zr produced in the frequency sweep mode and at the single frequencies of 62, 713 and 3137 Hz.

The results of the potentiodynamic polarisation tests are shown in Figure 12. Tafel slopes are indicated for the cathodic part of the PDP curve, which was used for the *i_corr_* evaluation. The PDP curve for the uncoated substrate is located at lower potentials than those for the coated samples; this indicates significant passivation of the coated surface. Moreover, the corrosion currents *i_corr_*, which shift of the PDP curve to the left, appear to be much less for the coated surface. The *E_corr_* values of the coated samples vary within the experimental error, whereas the corrosion currents *i_corr_* appear to be different due to varying morphology between the samples obtained at different frequencies.

**Figure 13:**
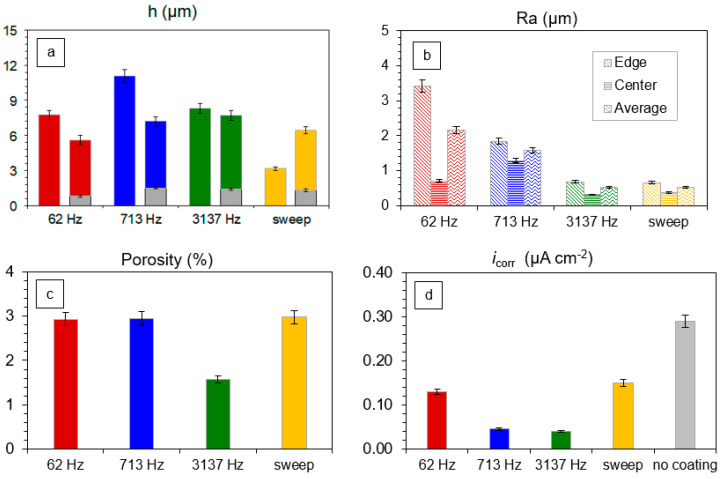
Coating thickness, *h* (**a**), roughness, *Ra* (**b**), porosity, *P* (**c**) and corrosion current density, *i_corr_* (**d**) for Zr samples after PEO treatments in the frequency sweep mode and at the constant frequencies of 62, 713 and 3137 Hz.

The coating thickness *h*, surface roughness *Ra*, porosity *P* and corrosion current, *i*_corr_, are compiled in Figure 13. In the coating thickness plot (Figure 13a), the left-hand-side bars belong to the measurements made with the eddy current gauge, and the right-hand-side bars belong to the estimates from the coating cross-sections (see Figure 11).

As the cross-section helped to identify the coating layers and their corresponding thicknesses, the right-hand-side bars have grey basements depicting the thickness of the inner layer, and the coloured bars stand for the thickness of the outer layer. The difference in the measurements can be attributed to the uniformity of the coating. The most uniform coating and the closest match between the measurements corresponds to the 3137 Hz experiment. The thickest coating was produced at 3137 Hz, whereas the frequency-swept experiment resulted in the thinnest coating.

The surface roughness at the sample edge and the average roughness notably decrease with the frequency; this demonstrates the degree of the edge effect. The roughness in the sample centre follows the coating thickness measured with the eddy current gauge. The coating produced at 3137 Hz possesses the lowest porosity; the values for the other regimes appear to be at the same level within the experiment error. The corrosion current decreases with frequency and generally follows the pattern of the coating porosity and average roughness. All the coated samples have a corrosion current lower than that of the substrate; the frequency-swept regime exhibits the values of the worst case, i.e., 62 Hz.

Compared to the results obtained elsewhere [27], we observe a significant influence of the electrolyte system on the formation of the coatings with the best protective properties under PUP PEO conditions. For the phosphate electrolyte system, the minimal porosity and the best coating properties correspond to lower frequencies (50 Hz), and maximal porosity appears at higher frequencies (1000 Hz) [27]. In contrast, in this study, the best coating properties were obtained at higher frequencies; therefore, the recommendations regarding the optimal frequency for the PUP PEO of Zr should be limited to the selected electrolyte system.

As a result, the frequencies in the vicinity of 3 kHz can contribute to the most efficient protective properties for the PEO coatings on zirconium obtained in the pulsed unipolar polarisation regime. At the same time, the application of the diagnostic frequency sweep should be minimised, as this provides a detrimental effect on the coating properties by the action of the microdischarges at lower frequencies.

### 3.4. Transient Analysis of the Voltage Pulses during the PUP PEO Process

**Figure 14:**
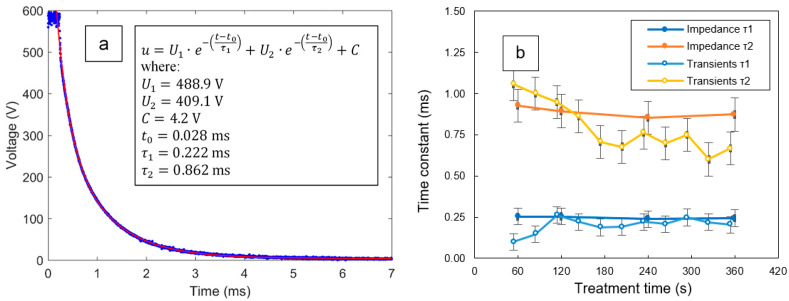
An example of fitting the voltage pulse transient with Equation (4) (**a**) and estimates of corresponding time constants τ_1_ and τ_2_ (**b**) performed using data of in situ impedance spectroscopy and pulse transient analysis.

Both time constants τ_1_ and τ_2_ characterizing the EC can be independently assessed from the voltage fall at the back edge of the pulse using Equation (2). These time constants are shown in Table 2 and represent the cutoff frequencies *f*_1_ and *f*_2_ at which the resistance *R* in an *R*–*C* pair is equal to the reactance 1/(2π*fC*). As the microdischarges disappear after the pulse, the observed voltage transient reflects discharging capacitances *C*_1_ and *C*_2_ via resistances *R*_1_ and *R*_2_. The corresponding transient is easy to observe in the waveforms. The resulting approximations have high goodness of fit (Figure 14a). The values of the time constants τ_1_ and τ_2_ obtained by fitting the voltage transients and in situ impedance spectra closely match each other (Figure 14b). The corresponding cutoff frequencies are consistent with the characteristic parts of the phase angle plot. For the lower-frequency loop of the impedance spectra, the cutoff frequency belongs to the range from 100–200 Hz; at these frequencies, the phase angle should have the maximum value, and this can be seen in Figure 8. For the higher-frequency loop, the cutoff frequency falls between 500 and 3000 Hz; this is consistent with Figure 8. As the *R*–*C* loops have resonant quality factors that are quite low, the maxima belonging to the two cutoff frequencies do not appear distinct.

### 3.5. Thickness and Morphology of the PEO Coating as a Function of Time

The following physical interpretation can be proposed for the elements of EC used to fit the in situ impedance spectra (Table 1). The electrolyte resistance is represented by the uncoupled element, *R_s_*. The branch *R*_2_–*C*_2_ corresponds to the inner coating layer of the PEO coating. The physical interpretation of *R*_2_ is the charge transfer resistance, and *C*_2_ is the effective capacitance. The branch *R*_1_–*C*_1_ corresponds to the outer porous part of the PEO coating which provides conductive pathways to the barrier layer. The physical interpretation of *R*_1_ is the charge transfer resistance through the outer layer, and *C*_1_ is the effective capacitance of this layer. As follows from Table 1, the values of *C*_1_ are an order of magnitude lower than that of *C*_2_; therefore, *C*_1_ stands for the thicker layer.

The equivalent thickness geometrical barrier *d* associated with capacitance *C* can be evaluated as

(6) $$d=\frac{\varepsilon_{\;}\varepsilon_{0}}{C}$$

using values of the dielectric constant for monoclinic zirconia ε = 13–23 [44]. The values of *d*_2_ range from 0.5 to 0.8 µm (Table 2), which is in relatively good agreement with the thickness of the inner barrier layer in the coating cross-section in Figure 11. For the outer layer, values of *d*_1_ range from 3.5 to 5.5 µm, which is consistent with the coating thickness measurements from the cross-sectional images.

Therefore, the proposed equivalent circuit is physically justified and can be used for in situ estimation of the thickness of the PEO coating layers.

### 3.6. Implications of the Frequency Response on the PUP PEO Process Optimization

Based on Figure 5 and Figure 13 and the video of the frequency-swept PUP PEO process, we can conclude that at frequencies from 2 to 5 kHz, the coating grows uniformly to almost the highest detected thickness; it has minimal roughness, porosity and corrosion current. This combination of the functional properties appears due to the uniform microdischarge distribution during the PEO process. This frequency range is characterized by a local minimum in the phase angle (see Figure 6). Even though the higher frequencies were disregarded from the estimation of the EC parameters, the impedance *Z_hf_* exists, and it has a resistive-capacitive type according to the experimental data in Figure 6. The investigation of this impedance appears to be outside the scope of this study, as the PEO equipment must be capable of providing higher-frequency technological pulses and must measure the electrical response with acceptable accuracy; this equipment has yet to be designed and produced. Nevertheless, the local minimum in the phase angle at frequencies from 2 to 5 kHz was experimentally detected.

**Figure 15:**
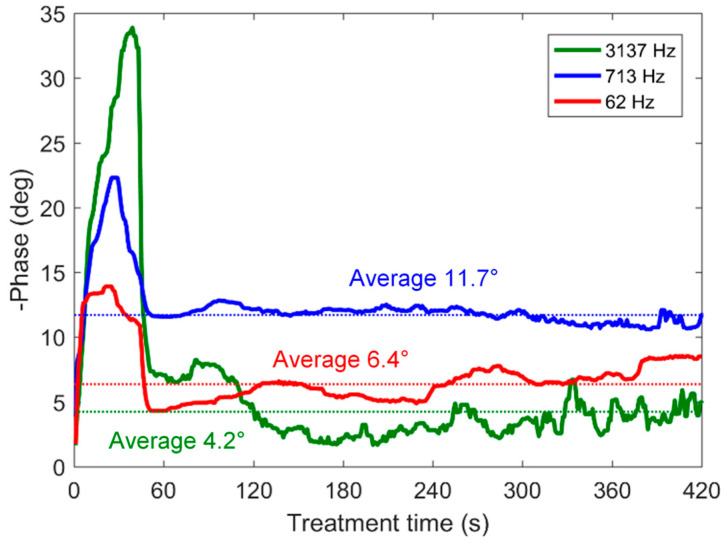
Phase shift between the voltage and current for the PEO of Zr at the single frequencies of 62, 713 and 3137 Hz.

This analysis is supported by Figure 15, which shows the variation of the phase angle θ for the single-frequency experiments. The PEO process carried out at 3137 Hz has the lowest phase angle after 2 min of the treatment following the soft start voltage ramp, while the process at 713 Hz has the highest θ; this frequency is close to the cutoff frequency *f*_1_, which corresponds to the maximal phase angle. This is consistent with the modelled phase angle of the impedance spectra (Figure 8).

A minimal phase angle at the frequencies not close to zero means that the power factor of such an electrical process is maximal, such that all the electrical energy is consumed into the resistance contributing to the energy transformations (from electrical energy into heat energy and the energy of chemical bonds); the reactance which reflects charging and discharging the capacitances appears to be minimal there. PEO treatments at frequencies below the minimum cutoff frequency result not only in close to a zero phase angle but also in the formation of coatings with a fused morphology at the sample edges as the pulse voltage falls to zero, and microdischarges need to be reignited at every pulse. This reignition follows the current density distribution in the electrolyte, which has the maximal local current densities at the sample edges; therefore, the reignition of the microdischarges appears at the edges, and the edges show a burned-coating morphology.

As a result, the PUP PEO process in the frequency range from 2 to 5 kHz and in the selected electrolyte provides uniform microdischarge distribution over the surface of the zirconium sample, which results in uniform coating growth at a power factor close to the unity. The optimum combination of processing parameters for the PUP PEO of Zr established by in situ impedance spectroscopy can support the further development of smart electrolytic plasma technologies for the protection of Zr alloys in various applications; the swept diagnostic regime indeed helps to uncover the most efficient frequencies and justifies their application.

## 4. Conclusions

This study discusses the frequency effect on the PEO treatment of Zr in the pulsed unipolar polarisation regime and the resultant coating properties. The in situ impedance spectroscopy method was used and correlated with the results of the transient analysis and microdischarge video imaging.

- The frequency response of the PUP plasma electrolytic oxidation of Zr in an alkaline phosphate-silicate electrolyte comprises two relaxation processes with time constants of 240–250 µs and 850–930 µs and cutoff frequencies of 620–650 Hz and 170–190 Hz, which correspond to the charge transfer through the coating layers.
- A second-order ladder RC equivalent circuit was justified for this PEO process, and the circuit parameters were evaluated in the practically important frequency range from 20 Hz to 4 kHz.
- Using the capacitance values *C*_1_ and *C*_2_, two geometrical dielectric barriers were evaluated: a thinner 0.5–1 µm inner layer of the coating and a thicker 4–6 µm outer layer; these estimates are in agreement with the coating cross-sectional morphology.
- For the PUP PEO of Zr in an alkaline phosphate-silicate electrolyte, the optimal coatings showing minimal porosity and the lowest corrosion currents correspond to the reactance of the minimal in situ PEO process; this reactance appears in the frequency range from 2–5 kHz where the PEO electric regime operates at the highest power factor (efficiency).
- For the PUP PEO of other substrates, similar optimal conditions can be identified using frequency-swept polarisation, either in a separate experiment or incorporated as a diagnostic signal in the working polarisation waveform.

Finally, the results of this study can contribute to the development of smart plasma electrolytic technologies featuring process optimization and online control of unobservable surface characteristics for the production of protective coatings on zirconium and other valve metals.

## Figures and Tables

**Table 1:** Equivalent circuit element values for the fitting of the in situ impedance spectra of Zr plasma electrolytic oxidation.

Treatment Time (min)	*R_s_* (Ω·cm^2^)	*Z_hf_* (Ω·cm^2^)	*R*_1_ (Ω·cm^2^)	*C*_1_ (µF·cm^−2^)	*R*_2_ (Ω·cm^2^)	*C*_2_ (µF·cm^−2^)
1	130.0 ± 10.0	365.1 ± 34.4	475.1 ± 94.3	0.533 ± 0.123	253.1 ± 104.0	3.66 ± 3.13
2	130.0 ± 10.0	521.4 ± 34.9	598.5 ± 92.5	0.425 ± 0.084	305.2 ± 104.5	2.93 ± 1.58
4	130.0 ± 10.0	590.2 ± 34.4	632.3 ± 86.1	0.377 ± 0.071	324.6 ± 99.3	2.63 ± 1.14
6	130.0 ± 10.0	604.3 ± 30.8	655.0 ± 86.9	0.372 ± 0.066	338.4 ± 99.7	2.58 ± 1.07

**Table 2:** Time constants, cutoff frequencies and thickness of the coating layers estimated from the in situ impedance spectra of Zr plasma electrolytic oxidation.

Treatment Time (min)	*τ*_1_ (ms)	*τ*_2_ (ms)	*f*_1_ (Hz)	*f*_2_ (Hz)	*d*_1_ (µm)	*d*_2_ (µm)
1	0.253 ± 0.051	0.926 ± 0.103	629 ± 8	172 ± 6	3.82 ± 0.61	0.56 ± 0.11
2	0.254 ± 0.053	0.894 ± 0.110	626 ± 8	178 ± 6	4.79 ± 0.52	0.70 ± 0.09
4	0.238 ± 0.049	0.854 ± 0.098	668 ± 7	187 ± 5	5.40 ± 0.51	0.77 ± 0.10
6	0.244 ± 0.048	0.873 ± 0.092	654 ± 7	182 ± 4	5.47 ± 0.60	0.79 ± 0.12

## Data Availability

The link to a typical video fragment of a frequency-swept PEO: https://www.youtube.com/watch?v=f5L2pdGycuI (accessed on 16 December 2023). Other data can be obtained from the corresponding author on request.

## References

[1] Duan Z., Yang H., Satoh Y., Murakami K., Kano S., Zhao Z., Shen J., Abe H. (2017). Current status of materials development of nuclear fuel cladding tubes for light water reactors. Nucl. Eng. Des..

[2] Motta A.T., Capolungo L., Chen L.Q., Cinbiz M.N., Daymond M.R., Koss D.A., Lacroix E., Pastore G., Simon P.C.A., Tonks M.R. (2019). Hydrogen in zirconium alloys: A review. J. Nucl. Mater..

[3] Wang Y.M., Feng W., Xing Y.R., Ge Y.L., Guo L.X., Ouyang J.H., Jia D.C., Zhou Y. (2018). Degradation and structure evolution in corrosive LiOH solution of microarc oxidation coated Zircaloy-4 alloy in silicate and phosphate electrolytes. Appl. Surf. Sci..

[4] Jiang G., Xu D., Yang W., Liu L., Zhi Y., Yang J. (2022). High-temperature corrosion of Zr–Nb alloy for nuclear structural materials. Prog. Nucl. Energy.

[5] Pieralli S., Kohal R.-J., Lopez Hernandez E., Doerken S., Spies B.C. (2018). Osseointegration of zirconia dental implants in animal investigations: A systematic review and meta-analysis. Dent. Mater..

[6] Lijnev A., Elango J., Gomez-Lopez V.M., Perez-Albacete Martinez C., Granero Marin J.M., Mate Sanchez De Val J.E. (2022). Antibacterial and Proliferative Effects of NaOH-Coated Titanium, Zirconia, and Ceramic-Reinforced PEEK Dental Composites on Bone Marrow Mesenchymal Stem Cells. Pharmaceutics.

[7] Xue R., Wang D., Yang D., Zhang L., Xu X., Liu L., Wu D. (2020). Novel Biocompatible Zr-Based Alloy with Low Young’s Modulus and Magnetic Susceptibility for Biomedical Implants. Materials.

[8] Stojadinović S., Tadić N., Vasilić R. (2018). Down-conversion photoluminescence of ZrO2:Er3+ coatings formed by plasma electrolytic oxidation. Mater. Lett..

[9] Trivinho-Strixino F., Guimarães F.E.G., Pereira E.C. (2008). Zirconium oxide anodic films: Optical and structural properties. Chem. Phys. Lett..

[10] Attarzadeh N., Ramana C.V. (2021). Plasma Electrolytic Oxidation Ceramic Coatings on Zirconium (Zr) and ZrAlloys: Part I—Growth Mechanisms, Microstructure, and Chemical Composition. Coatings.

[11] Lin J., Li H., Szpunar J.A., Bordoni R., Olmedo A.M., Villegas M., Maroto A.J.G. (2004). Analysis of zirconium oxide formed during oxidation at 623 K on Zr-2.5Nb and Zircaloy-4. Mater. Sci. Eng. A.

[12] Sidelev D.V., Kashkarov E.B., Syrtanov M.S., Krivobokov V.P. (2019). Nickel-chromium (Ni–Cr) coatings deposited by magnetron sputtering for accident tolerant nuclear fuel claddings. Surf. Coat. Technol..

[13] Cheol Lee G., Noh H., Yeom H., Jo H., Kyun Kim T., Kim M., Sridharan K., Sun Park H. (2019). Zirconium-silicide coating on zircaloy-4 substrate for accident tolerance: Effects on oxidation resistance and boiling. Ann. Nucl. Energy.

[14] Xiao W., Chen H., Liu X., Tang D., Deng H., Zou S., Ren Y., Zhou X., Lei M. (2019). Thermal shock resistance of TiN-, Cr-, and TiN/Cr-coated zirconium alloy. J. Nucl. Mater..

[15] Lorenzo-Martin C., Ajayi O.O., Hartman K., Bhattacharya S., Yacout A. (2019). Effect of Al_2_O_3_ coating on fretting wear performance of Zr alloy. Wear.

[16] Cheng Y., Matykina E., Arrabal R., Skeldon P., Thompson G.E. (2012). Plasma electrolytic oxidation and corrosion protection of Zircaloy-4. Surf. Coat. Technol..

[17] Apelfeld A.V., Borisov A.M., Krit B.L., Ludin V.B., Polyansky M.N., Romanovsky E.A., Savushkina S.V., Suminov I.V., Tkachenko N.V., Vinogradov A.V. (2015). The study of plasma electrolytic oxidation coatings on Zr and Zr-1% Nb alloy at thermal cycling. Surf. Coat. Technol..

[18] Malinovschi V., Marin A., Negrea D., Andrei V., Coaca E., Mihailescu C.N., Lungu C.P. (2018). Characterization of Al_2_O_3_/ZrO_2_ composite coatings deposited on Zr-2.5Nb alloy by plasma electrolytic oxidation. Appl. Surf. Sci..

[19] Attarzadeh N., Ramana C.V. (2021). Plasma Electrolytic Oxidation Ceramic Coatings on Zirconium (Zr) and Zr-Alloys: Part-II: Properties and Applications. Coatings.

[20] Cengiz S., Uzunoglu A., Huang S.M., Stanciu L., Tarakci M., Gencer Y. (2021). An in-vitro study: The effect of surface properties on bioactivity of the oxide layer fabricated on Zr substrate by PEO. Surf. Interfaces.

[21] Sowa M., Simka W. (2018). Effect of DC Plasma Electrolytic Oxidation on Surface Characteristics and Corrosion Resistance of Zirconium. Materials.

[22] Walsh F.C., Low C.T.J., Wood R.J.K., Stevens K.T., Archer J., Poeton A.R., Ryder A. (2009). Plasma electrolytic oxidation (PEO) for production of anodised coatings on lightweight metal (Al, Mg, Ti) alloys. Trans. Inst. Met. Finish..

[23] Clyne T.W., Troughton S.C. (2018). A review of recent work on discharge characteristics during plasma electrolytic oxidation of various metals. Int. Mater. Rev..

[24] Hussein R.O., Nie X., Northwood D.O. (2016). Production of high quality coatings on light alloys using Plasma Electrolytic Oxidation (PEO). WIT Trans. Built Environ..

[25] Cengiz S., Uzunoglu A., Stanciu L., Tarakci M., Gencer Y. (2016). Direct fabrication of crystalline hydroxyapatite coating on zirconium by single-step plasma electrolytic oxidation process. Surf. Coat. Technol..

[26] Gencer Y., Tarakci M., Cengiz S., Gunduz K.O. (2012). The Effect of Sodium Silicate Concentration on the Properties of the Coating Formed on Pure Zirconium by Microarc Oxidation Coating Technique. Adv. Mater. Res..

[27] Sandhyarani M., Ashfaq M., Arunnellaiappan T., Selvan M.P., Subramanian S., Rameshbabu N. (2015). Effect of electrical parameters on morphology and in-vitro corrosion resistance of plasma electrolytic oxidized films formed on zirconium. Surf. Coat. Technol..

[28] Fatimah S., Kamil M.P., Kwon J.H., Kaseem M., Ko Y.G. (2017). Dual incorporation of SiO_2_ and ZrO_2_ nanoparticles into the oxide layer on 6061 Al alloy via plasma electrolytic oxidation: Coating structure and corrosion properties. J. Alloys Compd..

[29] Krit B.L., Apelfeld A.V., Borisov A.M., Morozova N.V., Rakoch A.G., Suminov I.V., Grigoriev S.N. (2023). Plasma Electrolytic Modification of Zirconium and Its Alloys: Brief Review. Materials.

[30] Savushkina S., Gerasimov M., Apelfeld A., Suminov I. (2021). Study of Coatings Formed on Zirconium Alloy by Plasma Electrolytic Oxidation in Electrolyte with Submicron Yttria Powder Additives. Metals.

[31] Parfenov E.V., Yerokhin A. (2011). Methodology of data acquisition and signal processing for frequency response evaluation during plasma electrolytic surface treatments. Process Control: Problems, Techniques and Applications.

[32] Wang S., Liu Y.C., Liu H.M., Lan Y.F. (2023). In-situ estimate of coating by equivalent circuit for PEO of AZ31B. Surf. Eng..

[33] Mohedano M., Lopez E., Mingo B., Moon S., Matykina E., Arrabal R. (2022). Energy consumption, wear and corrosion of PEO coatings on preanodized Al alloy: The influence of current and frequency. J. Mater. Res. Technol..

[34] Barsukov E., Macdonald J.R. (2005). Impedance Spectroscopy: Theory, Experiment, and Applications.

[35] Dilimon V.S., Shibli S.M.A. (2023). A Review on the Application-Focused Assessment of Plasma Electrolytic Oxidation (PEO) Coatings Using Electrochemical Impedance Spectroscopy. Adv. Eng. Mater..

[36] Tsai D.-S., Chen G.-W., Chou C.-C. (2019). Probe the micro arc softening phenomenon with pulse transient analysis in plasma electrolytic oxidation. Surf. Coat. Technol..

[37] Yerokhin A., Parfenov E.V., Matthews A. (2016). In situ impedance spectroscopy of the plasma electrolytic oxidation process for deposition of Ca- and P-containing coatings on Ti. Surf. Coat. Technol..

[38] Dehnavi V., Luan B.L., Liu X.Y., Shoesmith D.W., Rohani S. (2015). Correlation between plasma electrolytic oxidation treatment stages and coating microstructure on aluminum under unipolar pulsed DC mode. Surf. Coat. Technol..

[39] Parfenov E.V., Yerokhin A., Matthews A. (2009). Small signal frequency response studies for plasma electrolytic oxidation. Surf. Coat. Technol..

[40] Farrakhov R.G., Mukaeva V.R., Fatkullin A.R., Gorbatkov M.V., Tarasov P.V., Lazarev D.M., Ramesh Babu N., Parfenov E.V. (2018). Plasma electrolytic oxidation treatment mode influence on corrosion properties of coatings obtained on Zr-1Nb alloy in silicate-phosphate electrolyte. IOP Conf. Ser. Mater. Sci. Eng..

[41] Tu W., Zhu Z., Zhuang X., Cheng Y., Skeldon P. (2019). Effect of frequency on black coating formation on AZ31 magnesium alloy by plasma electrolytic oxidation in aluminate-tungstate electrolyte. Surf. Coat. Technol..

[42] Yerokhin A., Parfenov E.V., Liang C.J., Mukaeva V.R., Matthews A. (2013). System linearity quantification for in-situ impedance spectroscopy of plasma electrolytic oxidation. Electrochem. Commun..

[43] Chen Q., Lei M., Chen Y., Deng Y., Chen M.A. (2023). Preparation of a thick sponge-like structured amorphous silica ceramic coating on 6061 aluminum alloy by plasma electrolytic oxidation in TEOS solution. Ceram. Int..

[44] Thompson D.P., Dickins A.M., Thorp J.S. (1992). The dielectric properties of zirconia. J. Mater. Sci..

